# Efficacy of Commercially Available Entomopathogenic Agents against the Polyphagous Shot Hole Borer in South Africa

**DOI:** 10.3390/insects14040361

**Published:** 2023-04-05

**Authors:** Wilma J. Nel, Bernard Slippers, Michael J. Wingfield, Neriman Yilmaz, Brett P. Hurley

**Affiliations:** 1 Department of Zoology and Entomology, Forestry and Agricultural Biotechnology Institute, University of Pretoria, Private Bag X20, Hatfield, Pretoria 0028, South Africa; 2 Department of Biochemistry, Genetics and Microbiology, Forestry and Agricultural Biotechnology Institute, University of Pretoria, Private Bag X20, Hatfield, Pretoria 0028, South Africa

**Keywords:** *Bacillus thuringiensis*, *Beauveria bassiana*, biological control, *Euwallacea fornicatus*, *Metarhizium anisopliae*

## Abstract

**Simple Summary:**

The accidental introduction of the ambrosia beetle *Euwallacea fornicatus* and its fungal symbiont, *Fusarium euwallaceae*, into Israel, the United States and South Africa has had a devastating impact on many economically and ornamentally important tree species. Currently, there is no effective control measure in place to control this beetle pest and its symbiont. As a first step towards controlling this beetle in South Africa, this study investigated the efficacy of commercialized entomopathogenic products against the beetle. Results showed that although effective under lab conditions, currently recommended field application methods of these products to the outer bark of trees have limited effects on beetle survival and reproduction.

**Abstract:**

The invasive ambrosia beetle, *Euwallacea fornicatus*, was first reported in South Africa in 2018. The beetle has now spread to eight provinces of the country and has had a devastating impact on both native and non-native tree species. This is especially true for trees located in urban and peri-urban environments. Recent predictions are that the South African *E. fornicatus* invasion will cost an estimated ZAR 275 billion (approx. USD 16 billion) if it continues to spread uncontrollably, justifying an urgent need for its effective management in the country. One option is biological control, which is preferred over the use of chemicals due to its lower environmental impact. We tested two broad-spectrum fungal entomopathogenic agents, Eco-Bb^®^ and Bio-Insek, which are commercially available in South Africa, for efficacy against *E. fornicatus*. Initial laboratory assays yielded promising results. However, beetle infestation trials using treated pieces of woody castor bean stems showed little effect on beetle survival and reproduction.

## 1. Introduction

*Euwallacea fornicatus* (Eichhoff, 1868), commonly known as the polyphagous shot hole borer (PSHB), is a small ambrosia beetle (Coleoptera: Curculionidae: Scolytinae) residing in the Xyleborini [1]. Ambrosia beetles such as the PSHB live in tunnel systems (galleries) deep in the sapwood of trees in association with obligate nutritional symbiotic ambrosia fungi [2]. Despite often being benign in the native ranges of their beetle hosts, ambrosia fungi can be plant pathogens, as is true for the symbiont of the PSHB, *Fusarium euwallaceae* [3,4]. The accidental introduction of the PSHB and its symbiont into Israel, the USA and South Africa has garnered considerable attention due to the extensive losses that they are causing to trees of economic and ornamental importance [5].

The first official report of PSHB in South Africa was published in 2018, although there is evidence that the beetle has been in the country since at least 2012 [5,6]. It has subsequently spread to all but one province (Limpopo) in the country and has been confirmed to infest more than 100 tree species [5,7]. Although the beetle has been reported on some commercially valuable tree species, its effect in these situations has thus far been minimal [8,9,10,11]. In contrast, the beetle has been causing massive devastation in some urban settings. This is especially true in areas such as Johannesburg where the PSHB, together with its symbiont *F. euwallaceae*, is killing many old and well-established trees. If left unchecked, it is estimated that PSHB will result in economic losses of approximately ZAR 275 billion (approx. USD 16 billion) in the next 10 years, much of this necessitated by the responsible removal of some 65 million infested trees in urban environments [7].

The unique lifestyle of ambrosia beetles deep within trees makes these insects notoriously difficult to control [12,13,14]. In recent years, biological control options have become a promising alternative to chemical control strategies for ambrosia beetle management. Various studies have considered the use of entomopathogenic microbes as a means of control with varying degrees of success [12,13,15,16,17,18,19]. However, the development and registration of new biological control agents can be a costly and time-consuming process. Therefore, as the focus of this study, we decided to investigate potential biological control agents already registered for use in South Africa, speeding up the process of their implementation. As an initial step in PSHB management in South Africa, we tested two available broad-spectrum commercial entomopathogenic agents to determine whether they might be used as a preventative measure against PSHB, specifically to protect high-value trees in urban environments. Despite initial lab trials showing promising results, infestation trials were less promising. However, performing these trials allowed us to identify flaws in current methods of biological control application that are potentially problematic in the PSHB system.

## 2. Materials and Methods

### 2.1. Rearing of PSHB

Beetles were reared on a sawdust-based artificial diet as described by Cooperband et al. [20]. Ingredients were mixed in a 1 L glass Schott bottle and consisted of 75 g London plane (*Platanus* ×*acerifolia*) sawdust, 20 g agar, 10 g sucrose, 5 g casein, 5 g corn starch, 5 g yeast, 1 g Wesson’s salt mix, 0.35 g streptomycin, 2.5 mL wheat germ oil, 5 mL 96% ethanol and 500 mL ddH2O. Dry ingredients were mixed first before adding the wet ingredients and autoclaving for 25 min at 121 °C. Approximately 20 mL of diet was then poured into 50 mL polyethylene centrifuge tubes. The tubes were loosely capped and allowed to dry in a flow hood for 7–14 days, allowing all condensation to evaporate. The tubes were sealed and maintained at 4 °C until use.

Initial foundress beetles for rearing were collected from naturally infested oak logs (*Quercus* sp., Rietondale, Pretoria, South Africa, 2021). Logs were maintained in emergence chambers, and the emerging beetles were collected. Foundress beetles were surface disinfested in 70% ethanol for 10 s and allowed to air dry before being transferred to individual rearing tubes. Each rearing tube was capped with a ventilated lid and maintained in a plastic container in the laboratory. Following the establishment of the beetle colonies, the adult female progeny was transferred to fresh diet tubes every 8–10 weeks to maintain the population.

### 2.2. Entomopathogenic Agents and Laboratory Assays

Six different entomopathogenic fungal products were considered for screening against PSHB. Three of these products (Nomu-Protec (Andermatt group, Grossdietwil, Switzerland), Lecatech ^®^ WP and Mytech ^®^ WP(Both Dudutech, Naivasha, Kenya))) were excluded due to their limited host range. A fourth product, BroadBand ^®^ (BASF, Ludwigshafen, Germany), was further excluded due to it only being available for purchase at commercial scale and not quantities suitable for laboratory testing. The two remaining products, Bio-Insek (Agro-Organics, Somerset West. South Africa) and Eco-Bb ^®^(Andermatt group, Grossdietwil, Switzerland), were selected for screening against PSHB (Table 1) based on their broad host specificity, being registered for commercial application in South Africa and being easily accessible to members of the public.

Two laboratory assays were performed to assess the efficacy of these agents against the PSHB: (1) direct application of entomopathogen fungal suspension to beetles, and (2) application of entomopathogen fungal suspension to filter paper on which the beetles were maintained. Assays were incubated for 10 days in darkness at 23 °C without providing food.

In the first assay, 8-week-old female beetles were excavated from their rearing tubes. Female beetles were selected to be used in the assays as they are the dispersing sex and would be most likely to encounter the biological control agents. Fecundity of the female beetles was not determined prior to use in the assays. Each trial replicate consisted of eight beetles that were exposed to one of the entomopathogenic agents or a control treatment of sterile distilled water, and three replicates were performed per agent (n = 24 beetles per treatment). A suspension was prepared for each of the entomopathogenic agents (Table 1) by mixing 10 mL autoclaved ddH_2_O with 0.2 g Eco-Bb^®^ or 0.4 g Bio-Insek spore powder, respectively, scaled down from the instructions given on the product packaging. Suspensions were mixed thoroughly by vortexing for 1 min. From these suspensions, 1 mL was pipetted to evenly cover the surface of individual 35 mm Petri dishes. The beetles were then individually exposed to one of the treatments (Eco-Bb^®^, Bio-Insek, or control) for 1 min by allowing them to walk in the suspension [15]. Each of the eight beetles making up a single treatment replicate was then transferred to a 65 mm Petri dish containing a filter paper disk moistened with sterile water to maintain humidity. Beetles were inspected daily, and mortality was recorded.

In the second assay, spore suspensions of the entomopathogenic agents or sterile water to serve as a control were prepared as described previously, and 1 mL was used to impregnate 55 mm (ø) filter paper disks. Nine 65 mm Petri dishes were lined with the impregnated filter paper discs (3× Eco-Bb^®^, 3× Bio-Insek, and 3× control) and eight beetles were added per Petri dish (n = 24 beetles per treatment). Beetles were inspected daily, and mortality was recorded.

### 2.3. Beetle Infestation Assay

To assess the efficacy of the entomopathogenic agents during beetle boring, an infestation assay was performed using woody stem pieces of castor bean (*Ricinus cummunis*), a common reproductive host of the beetle in South Africa. Collected stems were sectioned into 40 pieces (12.1 cm × 3.3 cm (average length by diameter)) and were used within 24 h of collection. Both the Eco-Bb^®^ and Bio-Insek products were tested at two concentrations in the infestation assay. These included (1) the recommended dose as indicated by the manufacturer, and (2) 10 times (10×) the recommended concentration (Table 1). Eight pieces were inoculated per treatment with the entomopathogenic agents and eight were dipped in sterile water as a control ((8 × 4) + 8 = n40), using the method described by Carrillo et al. [15]. Briefly, 2 L of spore suspension was prepared in individual sterile containers following the manufacturer instructions on the product packaging to obtain the correct concentrations. Stem pieces were then dipped and swirled in the prepared spore suspensions for 10 s to ensure an even coating of spores. The stem pieces were allowed to air dry for four hours at 21 °C. Two pieces per treatment were then placed in plastic containers with ventilated lids, with four replications per treatment. Six beetles were released per container (n = 24 beetles per treatment). Containers were maintained in a walk-in incubator at 25 °C for two weeks after which destructive sampling was performed to recover the beetles.

### 2.4. Spore Viability Assay

To confirm viability of the spores contained within the product packaging throughout the time of the study, a viability assay was performed for each assay. After spore suspensions were prepared, 100 µL of suspension was pipetted onto the surface of a clean malt extract agar plate (MEA: 2% malt extract, 2% BD^TM^Difco^TM^ Agar, Biolab, Midrand, South Africa). The suspension was spread evenly over the surface of the agar plate using a sterilized spreader, and plates were incubated at 23 °C in the dark for 24 h. After 24 h, germinating spores were counted within a 1 cm^2^ block on the agar surface. Spores were considered viable if a germ tube longer than the spores’ width had emerged. Five replicates were prepared for each suspension for each assay.

### 2.5. Observation for Mycosis

Throughout the incubation periods of both the direct and indirect application assays, dead beetles were removed and transferred to individual moisture chambers to observe for mycosis. Cadavers were removed immediately upon discovery from the trial replicates to prevent transmission between specimens. Moisture chambers used for incubation were constructed using 2 mL Eppendorf tubes that contained a plug of cotton wool at the bottom and a plastic platform to support the insect cadaver [21]. Moisture chambers containing individual beetles were incubated at 23 °C in the dark, and humidity was maintained by adding autoclaved ddH_2_O to the cotton wool at the bottom of the tubes every couple of days. Once mycosis was observed, representative individuals from each trial replicate were selected for isolation of the emerging fungi onto MEA plates to confirm identity using morphological characters.

### 2.6. Data Analyses

For both laboratory assays, a Kaplan–Meier estimator [22] was used on cumulative data of replicates to determine differences in median survival time (or the time point at which the probability of survival is estimated to be 50%) for the beetles treated with the different entomopathogenic agents using the package ‘survival’ in R [23]. To determine if there were significant differences between the different entomopathogenic agents at the different concentrations tested in the infestation assay, various statistical analyses were performed using R (R Core Team 2017; https://www.R-project.org/, accessed on 28 January 2023). Initially, Shapiro–Wilk and Levene’s tests were performed to determine if the data were normally distributed and homogenous. Two of the three parameters (i.e., the number of beetles that had infested the castor bean stems per treatment and the number of beetles recovered alive per treatment) passed these tests and were found to be normally distributed and homogenous, whereas one parameter (i.e., the number of beetles recovered from each treatment) did not pass these tests. Where the parameters were normally distributed and homogenous, both a one-way ANOVA and a Kruskal–Wallis rank sum test [24] with an ad hoc Dunn multiple comparison test [25] were performed to assess the significance, and for the non-normally distributed parameter, only the latter test was performed.

## 3. Results

### 3.1. Pathogenicity in Laboratory Assays

**Figure 1:**
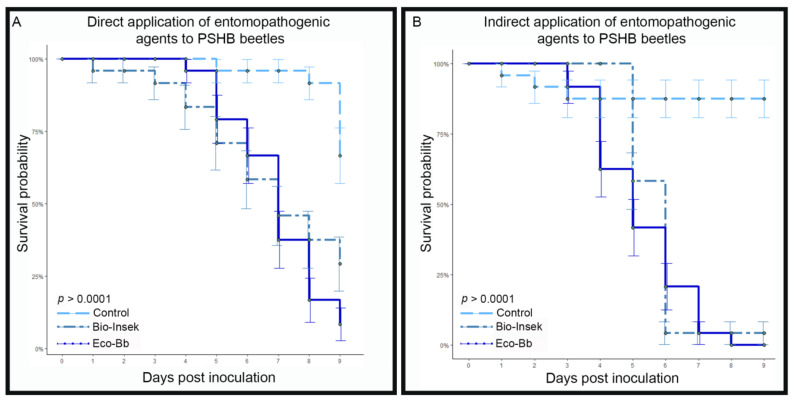
Kaplan–Meier survivorship curves reflecting time until death of PSHB beetles for the laboratory applications assays where (**A**) spore suspensions were applied directly to the beetles, and (**B**) spore suspensions were applied to the filter papers on which beetles were incubated. Beetles used in trial replicates were used as individual units to determine median survival time. Error bars indicate standard error, circles indicate the percentage of live beetles remaining at a given time point.

**Figure 2:**
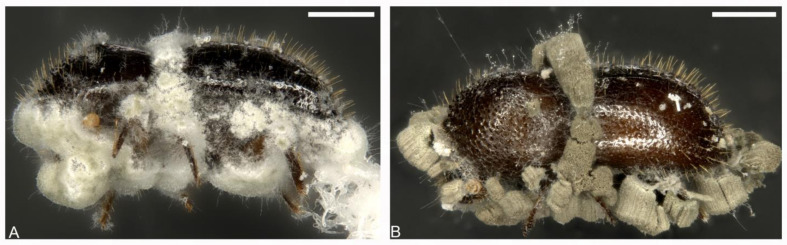
Mycosis observed in individual beetles from the direct application assay after continued incubation in moist chambers: (**A**) beetle inoculated with Eco-Bb^®^ showing *Beauvaria bassiana* growth, and (**B**) beetle inoculated with Bio-Insek showing *Metarhizium anisopliae* growth. Scale = 0.5 mm.

Direct application of spore suspensions of the products Eco-Bb^®^ and Bio-Insek both resulted in a reduced median survival time (MST) of PSHB beetles compared to the control treatments (Figure 1A). Both products had an MST of 7 days. MST was not reached by beetles treated with sterile water at 10 days post-inoculation. The indirect application assay again resulted in reduced MST of the beetles for both fungal products. Beetles incubated in the presence of filter paper disks treated with Eco-Bb^®^ reached MST 5 days post-introduction, and beetles incubated in the presence of filter paper disks treated with Bio-Insek reached MST 6 days post-introduction (Figure 1B). Beetles incubated on the control filter paper disks inoculated with sterile water did not reach MST before the end of the trial. Continued incubation of beetle cadavers from both trials in individual moisture chambers resulted in the emergence of mycosis resembling that of the inoculated EPFs (Figure 2). For beetles inoculated with suspensions of Eco-Bb^®^, typical *Beauveria bassiana* growth was observed on beetle cadavers (Figure 2A) and in culture. For beetles inoculated with suspensions of Bio-Insek, mycosis resembling *Metarhizium anisopliae* was seen on beetle cadavers (Figure 2B) and in culture.

### 3.2. Pathogenicity in Infestation Assays

**Figure 3:**
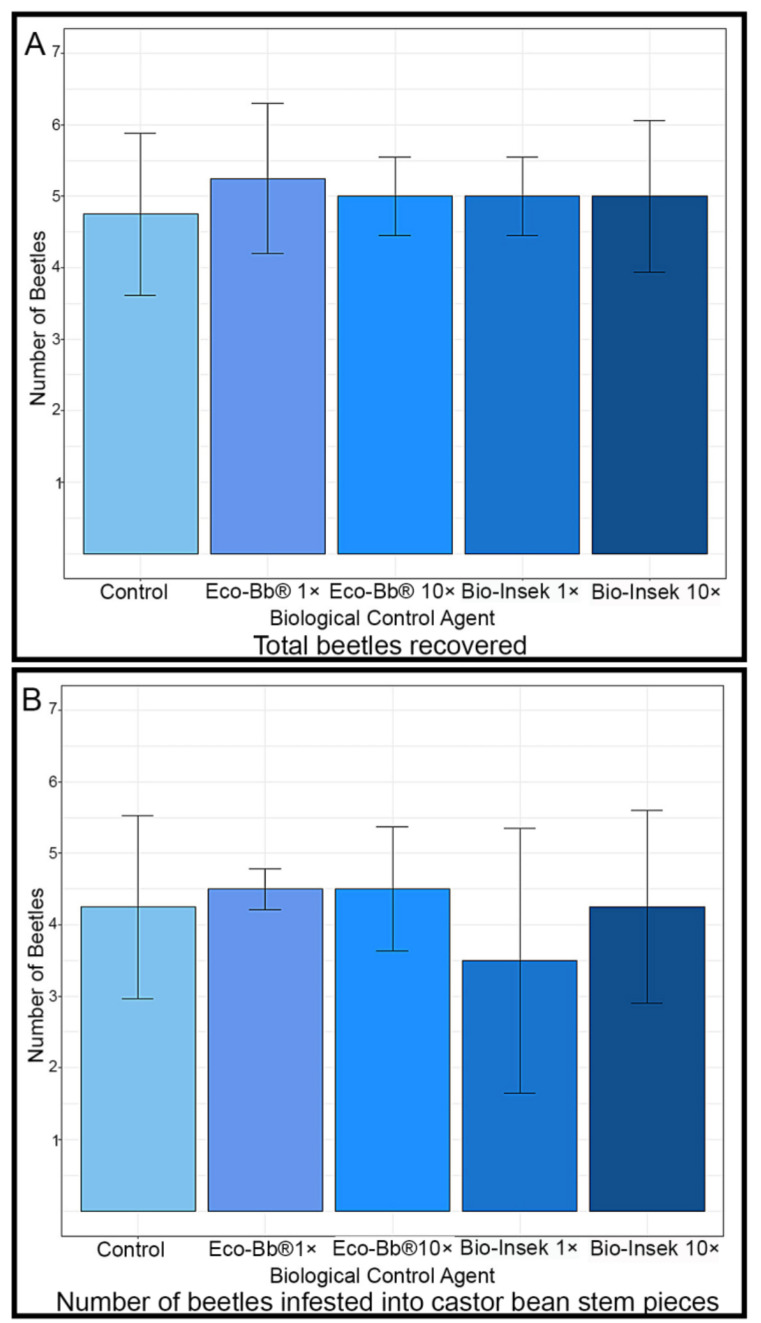

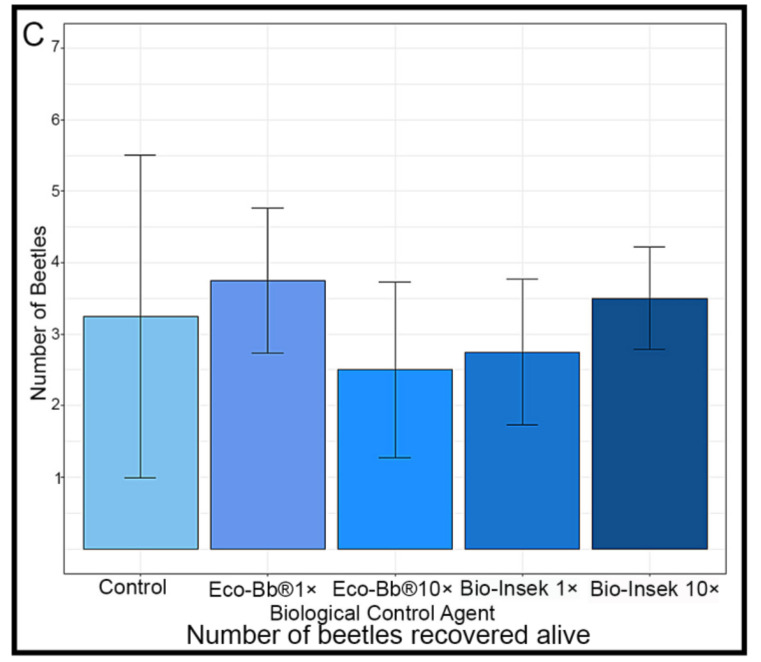
Bar charts showing (**A**) the average total number of beetles recovered, (**B**) the number of beetles that infested into the castor bean stems, and (**C**) the average number of beetles recovered alive from the trial replicates of the beetle boring assay. Error bars show plus or minus the standard error or the mean.

For each of the four respective fungal treatments and control treatment, comparable numbers of beetles were recovered/harvested from the castor bean stems (Figure 3A, χ^2^ = 0.54114, df = 4, *p*-value = 0.9694). Of the 24 initially introduced beetles, 19 beetles (79%) were recovered from the control treatment replicates, 21 beetles (88%) were recovered from the recommended dose Eco-Bb^®^ treatment replicates and 20 beetles (83%) were recovered for each of the three remaining treatments, i.e., Bio-Insek at recommended and 10× doses and the 10× dose of Eco-Bb^®^. The presence of the entomopathogenic fungi had no significant effect on the boring activities of the beetles, and similar numbers were found to have had infested the castor bean stems in all trial replicates (Figure 3B, χ^2^ = 0.80098, df = 4, *p*-value = 0.9383). The application of the entomopathogens to the castor bean stems also had little effect on beetle mortality, with similar numbers of living and dead beetles recovered from the different trials (Figure 3C, χ^2^ = 2.599, df = 4, *p*-value = 0.627).

**Figure 4:**
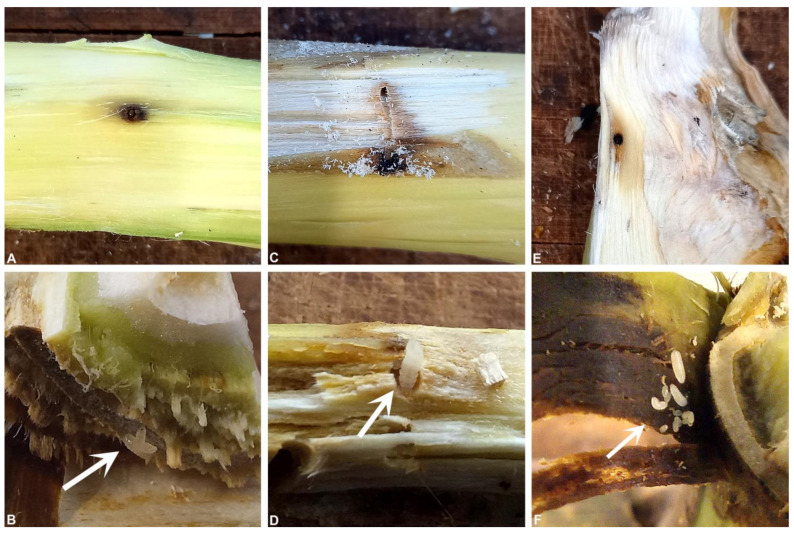
Beetles were able to infest (**A**,**C**,**E**) and reproduce (**B**,**D**,**F**) in castor bean stems treated with various doses of the entomopathogenic agents. Larvae (arrows) were found present in replicates of all treatments, excluding those of the recommended dose application of Bio-Insek. Shown here are replicates of control (**A**,**B**), 10× Eco-Bb^®^ (**C**,**D**) and 10× Bio-Insek (**E**,**F**) treatments.

No consistent patterns of mycoses (i.e., visible outward fungal growth of the entomopathogen on the beetle cadavers) were observed for beetles recovered from the infestation assay. Mycosis matching that of the inoculated fungal agents was observed for only four of the beetles recovered from the infested castor bean pieces. Two of the beetles were recovered from different Bio-Insek applications, one from a recommended dose replicate and one from a 10× dose replicate, each showing signs of *M. anisopliae* growth. Two additional beetles were recovered from different replications of the 10× Eco-Bb^®^ application showing signs of *B. bassiana* growth. However, none of these four beetles had infested the woody material. They had died in the container where they were exposed to high levels of inoculum present on the surface of the castor bean stems for the duration of the experiment. Living female beetles tending eggs and larvae were observed in several replicates, including those treated with 10× the recommended dose of the entomopathogenic agents (Figure 4). This suggested that neither of the entomopathogenic products had an effect on beetle reproduction.

## 4. Discussion

Laboratory assays investigating the efficacy of two entomopathogenic products that are commercially available in South Africa, Bio-Insek and Eco-Bb^®^, showed promise as preventative agents of PSHB. These products were able to reduce the survival of the beetles in both direct and indirect application assays compared to control treatments.

The MST values of the indirect application assays were lower in both products compared to the direct application assays. This is similar to results observed by Davis et al. 2018 [26], who also saw an increased MST on contact-treated surfaces. This is likely a result of the beetles being continuously exposed to high levels of viable inoculum, present on the surface of the filter paper disks, for the duration of incubation yielding higher rates of infection.

Despite initial success in the laboratory assays using the two fungal products, wood boring assays using woody castor bean stems treated with both the recommended dose of the products and 10 times this amount, had no significant effects on the survival of the beetles. There were no statistically significant differences between treatments for the beetles that infested the treated castor bean stems or the number of live beetles recovered. Signs of beetle reproduction were also seen in trial replicates, except those of the castor bean stems treated using the recommended dose of Bio-Insek. However, the recovery of living beetles from the material treated with both the recommended and 10× doses of Bio-Insek, and the presence of eggs and larvae seen in the 10× treated material, suggests that the beetles would be able to reproduce in this material after a longer incubation period.

In vitro application of fungal entomopathogens against infestation by bark and ambrosia beetles using application methods such as dipping [15,18,27], direct inoculation of the cuticle [26,28,29] and spraying [16,30,31] have all shown promising results. However, this initial success is often difficult to replicate under field conditions [32,33,34]. This is usually due to the sensitivity of these biological control agents to environmental conditions such as humidity, temperature and UV exposure [26,34]. The life strategy of ambrosia beetles adds an additional layer of complexity in their exposure to entomopathogenic agents. In the case of PSHB, females are the dispersing sex and will mate with their male siblings in the natal gallery before emerging in search of a new host [3]. Males will rarely venture outside their natal gallery. Therefore, the only opportunity to target the beetles for entomopathogen application is during the short dispersal period of the females. Unless a dispersing female is contaminated sufficiently before constructing her gallery, there is little chance of an entomopathogenic agent affecting her or her future brood. The environmental sensitivity of entomopathogenic fungi, coupled with their application to the outer bark of a host and short window of contamination opportunity, currently limits the use of these products to temporary preventatives for PSHB and would require continuous re-application to high-value trees.

## 5. Conclusions

Biological control has been used against invasive pests for more than a century. This is largely due to the lowered environmental risk offered and the potential for self-sustainability of pest management without a need for continued re-application. In this study, we tested two entomopathogenic agents commercially available in South Africa for their efficacy against the invasive ambrosia beetle PSHB. Despite promising initial results, infestation assays to determine their usefulness as preventative agents under more natural conditions showed little effect on beetle survival. To use entomopathogenic agents as a management strategy for PSHB, further work on the identification of more specific fungal strains adapted to the PSHB’s environment would be required, which could reduce the number of spores required to establish infection. Additionally, other modes of application should be considered, i.e., using spore dispersal traps, which could obtain higher rates of beetle exposure during the limited window of opportunity of a dispersing female. However, if these agents are to be used as more than just a preventative measure against the PSHB or other invasive ambrosia beetles, a method to actively deliver the agents into already established gallery systems will also be required.

## Figures and Tables

**Table 1:** Commercial entomopathogenic agents and the concentrations at which they were used in this study.

Registration Holder	Product	Active Organism	Concentration Used in Lab Assays	Concentrations Used in Infestation Assays	
				Recommended Concentration	10× Recommended Concentration
Agro-Organics	Bio-Insek	*Beauveria bassiana* (*Bb*) and *Metarhizium anisopliae* (*Ma*)	1.6 × 10^5^ spores/mL *Bb* 2.8 × 10^5^ spores/mL *Ma*	1.6 × 10^4^ spores/mL *Bb* 2.8 × 10^4^ spores/mL *Ma*	1.6 × 10^5^ spores/mL *Bb* 2.8 × 10^5^ spores/mL *Ma*
Plant Health Products	Eco-Bb^®^	*Beauveria bassiana*	4 × 10^6^ spores/ml	4 × 10^6^ spores/ml	4 × 10^7^ spores/ml

## Data Availability

Data is available from the authors upon reasonable request.
